# Evolution of Antibiotic Resistance without Antibiotic Exposure

**DOI:** 10.1128/AAC.01495-17

**Published:** 2017-10-24

**Authors:** Anna Knöppel, Joakim Näsvall, Dan I. Andersson

**Affiliations:** Department of Medical Biochemistry and Microbiology, Uppsala University, Uppsala, Sweden

**Keywords:** antibiotic resistance, Escherichia coli, Salmonella enterica, evolution, media adaptation

## Abstract

Antibiotic use is the main driver in the emergence of antibiotic resistance. Another unexplored possibility is that resistance evolves coincidentally in response to other selective pressures. We show that selection in the absence of antibiotics can coselect for decreased susceptibility to several antibiotics. Thus, genetic adaptation of bacteria to natural environments may drive resistance evolution by generating a pool of resistance mutations that selection could act on to enrich resistant mutants when antibiotic exposure occurs.

## TEXT

**FIG 1:**
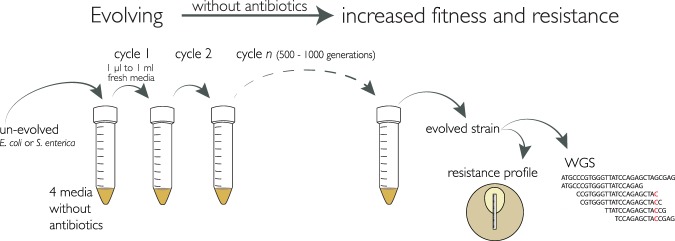
Experimental set-up. A total of 4 to 10 independent cultures of E. coli MG1655 and S. enterica subsp. enterica serovar Typhimurium LT2 were serially passaged for between 500 to 1,000 generations in four commonly used liquid growth media. The evolved populations were tested for antibiotic susceptibility by Etests and for genetic changes by whole-genome sequencing.

**FIG 2:**
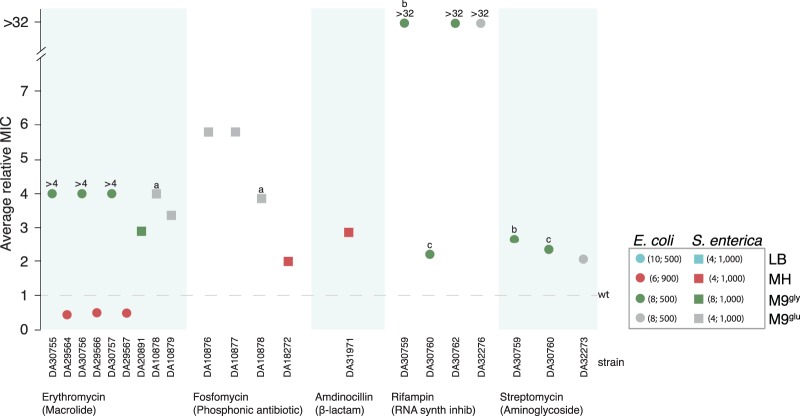
Altered susceptibility to antibiotics in bacterial populations evolved under antibiotic-free conditions. The media and species used are color-coded as depicted in the figure (LB, lysogeny broth; MH, Mueller-Hinton broth; M9, M9 minimal medium [12] supplemented with 0.2% glycerol [M9^gly^] or 0.2% glucose [M9^glu^]). The total number of evolved lineages (4 to 10) and generations (500 to 1,000) are indicated in the box. Labels a, b, and c indicate three evolved populations that each have decreased susceptibility to multiple antibiotics. Susceptibility to ampicillin, chloramphenicol, ciprofloxacin, nitrofurantoin, and tetracycline was also tested but no differences in susceptibility compared to the nonevolved wild types were found; in addition, no differences in susceptibility were found for lineages evolved in LB. Relative MIC values for populations that did not differ from wild type are not shown in the figure.

Since antibiotics came into widespread use some 70 years ago, the evolution and spread of antibiotic-resistant pathogens have been fueled by the extensive use and overuse of antibiotics in human and animals. Another factor, which may have been overlooked and which was studied here, is the presence of selective forces other than antibiotics that may cause accumulation of mutations that incidentally also confer decreased antibiotic susceptibility. Thus, selection for a specific cellular characteristic (for example, survival or growth under a specific condition) could yield pleiotropic effects in other parts of genetic/metabolic networks (1–4). Here we show that selection for growth medium adaptation mutations, i.e., mutations that increase growth rates in a specific growth medium, can result in decreased susceptibility to a number of different antibiotic classes. The study was performed by serial passage of 4 to 10 parallel lineages of wild-type Escherichia coli and Salmonella enterica strains for 500 to 1,000 generations in four different growth media lacking antibiotics (Fig. 1 and 2; see also Materials and Methods in the supplemental material). The evolved populations were tested with regard to their susceptibility to several classes of antibiotics (Etests) and whole-genome sequenced to identify potential contributing genetic changes. Unexpectedly, our findings show that antibiotic resistance can evolve in response to a novel selection pressure without any antibiotic exposure.

All of the 52 evolved populations (Fig. 1) were tested against 10 antibiotics from different classes, yielding 520 drug-population combinations. A substantial number of the lineages showed a significant increase (2 to >32-fold) in their MIC of different antibiotic classes. Thus, decreased susceptibility was observed against three different antibiotics (erythromycin, rifampin, and streptomycin) in 10 E. coli populations and against three different antibiotics (erythromycin, fosfomycin, and amdinocillin) in 8 S. enterica populations (Fig. 2). In total, 3.5% (18/520) of all tested combinations showed decreased susceptibility, whereas only 0.6% (3/520) showed increased susceptibility (three E. coli populations had a 2.3-fold reduction in the MIC of erythromycin). In all populations with decreased susceptibility, the putative resistance mutations (see below) showed signatures of selection, as the mutations had either gone to near-fixation or reached high frequencies in the population.

In all populations, between 1 and 10 mutations were identified (see Table S1 in the supplemental material), ranging in frequency between 10 to 100%. Little overlap in mutated genes was found between the two organisms and between different media, whereas extensive parallelism was sometimes seen for replicate populations grown in the same condition. Since each of the evolved populations with increased MIC contained more than one mutation that had reached a high frequency, a direct causality between specific mutations and altered susceptibility could not be established (Table S1). However, it is known from other studies that, for example, streptomycin, rifampin, and ciprofloxacin resistance can, in addition to the common resistance mechanisms (*rpsL*, *rpoB*, and *gyrA/B* mutations, respectively), also be conferred by mutations in metabolic functions (for example, electron transport and sugar metabolism) (5–7). Such mutations were indeed found in our evolved populations as shown by whole-genome sequencing (Table S1) and could potentially explain their decreased susceptibility. In line with this idea, a high fraction (approximately 20%) of the mutated genes in our evolved populations have previously been found to confer resistance to several different antibiotics and antimicrobial peptides (Table 1). Regarding RNA polymerase mutations, we found the *rpoB* (H526Y) mutation, which is known to lead to rifampin resistance and has repeatedly been selected for, both in the presence and absence of rifampin (1, 2, 4, 6, 8, 9). In addition, we found an amino acid substitution at position 1075 in *rpoC*, which has been described as both compensating for the cost of *rpoB* mutations and further increasing the MIC of rifampin (4). Furthermore, resistance to rifampin and nalidixic acid has been selected for in the absence of antibiotics in aging colonies (1) and *rpsL* mutations that confer resistance to streptomycin have been selected in media with poor carbon sources (3). It has been suggested that the fitness increase in RNA polymerase, *gyrA*, and *rpsL* mutants could be caused by altered RpoS expression or interaction with RNA polymerase and thus changes in bacterial stress responses (1, 3). It is plausible that our *rpoB* and *rpoC* mutations have similar effects.

**TABLE 1:** Genes mutated in our study that earlier have been described to confer resistance to antibiotics or antimicrobial peptides

Gene	Resistance	Reference(s)	Species
*cyaA*	Amdinocillin	13	E. coli
*envC*	Antimicrobial peptides	14	*E. coli, S. enterica*
*flu*	Chloramphenicol	15	E. coli
*ftsI*	β-Lactams	16	Haemophilus influenzae
*ftsI*^*a*^	Ertapenem, meropenem	17	E. coli
*ftsQ*	Amdinocillin	18	E. coli
*ftsX*	Chemokines, ceftriaxone	19, 20	Bacillus anthracis, Streptococcus pneumoniae, Neisseria gonorrhoeae
*mreC*	Amdinocillin	21	E. coli
*ompD*	Polymyxin B, cathelicidin^*c*^	22	S. enterica
*relA*	Vancomycin	23	Enterococcus faecalis
*rpoC*^*b*^	Rifampin	4	S. enterica
*rpsJ*	Tetracycline, tigecycline	24, 25	Acinetobacter baumannii, E. faecium, E. coli, N. gonorrhoeae, Staphylococcus aureus
*sapD/F*	Wheat α-thionin, snakin-1^*c*^	26	Erwinia chrysanthemi
*trkH*	Streptomycin	27	E. coli
*yciM*	Colistin	28	Klebsiella pneumoniae
*yodB*	Quinone	29	B. subtilis
*rpoB*	Streptolydigin, streptovaricin	30	Mycobacterium tuberculosis
*rpoB*^*b*^	Rifampin	1, 4, 7, 9	M. tuberculosis, S. enterica, S. aureus

a Confers resistance in combination with *envZ* mutations.

b Among others, the *rpoB* (H526Y) mutation and substitutions in the R1075 position in *rpoC* that were also found in this study.

c Antimicrobial peptides.

Why is decreased susceptibility relatively common (3.5%) but increased susceptibility (0.6%) comparatively rarer? A simple answer could be that in an evolving population, a gradient diffusion test (Etest) will easily detect subpopulations with decreased susceptibility (as some growth in the inhibition zone), whereas any mutant subpopulation with increased susceptibility would be hidden by less susceptible cells within the population. However, this is not a likely explanation here, since in all but three cases the inhibition zones were distinct and showed no indications of subpopulations or heterogeneity. A second potential explanation is that mutations that increase fitness in a medium could also lead to decreased susceptibility, simply because the bacteria are generally more fit. We cannot exclude this possibility but we find it less likely as generally the opposite is observed, i.e., a lower growth rate makes bacteria less susceptible to antibiotics (10). Another more interesting explanation is that there is no strict border between resistance mutations and medium adaptation mutations. Thus, mutations with global regulatory effects could cause both increased fitness and decreased antibiotic susceptibility by pleiotropic mechanisms. The *rpoB* (H526Y) mutation serves as an illustrative example: it is a known rifampin resistance mutation, but has also been shown to confer increased fitness under long-term starvation (in the absence of rifampin) (1). Similarly, other *rpoB* mutations have been shown to cause upregulation of a multidrug efflux pump, resulting in decreased susceptibility to antibiotics from different classes (11).

In conclusion, the high frequency of decreased susceptibility to different antibiotics in the populations evolved in the absence of antibiotics suggests that selection for one trait (increased fitness in a specific growth medium) may result in pleiotropic effects with regard to other traits. Such trade-offs have been observed in numerous other studies but the one observed here is of special interest, since it generated antibiotic resistance. A significant implication of these findings is that the continuous genetic adaptation of bacteria to various growth conditions in natural environments and hosts might serve as a driver of resistance evolution by generating standing genetic variation of resistance mutations that selection could act on to enrich resistant mutants when antibiotic exposure does occur.

## Supplementary Material

Supplemental material
