# Supplementary material for: Evolution of Antibiotic Resistance without Antibiotic Exposure
Source: Antimicrob Agents Chemother. 2017 Oct 24;61(11):e01495-17. doi: 10.1128/AAC.01495-17 (PMC5655081; doi:10.1128/AAC.01495-17)
Supplement: Supplemental material [file supp_61_11_e01495-17__index.html]

Supplemental material 

# Evolution of Antibiotic Resistance without Antibiotic Exposure

## Supplemental material

- Supplemental file 1 -

  Table S1 and Supplemental Materials and Methods

  PDF, 177K
