# Supplementary material for: Evolution of Antibiotic Resistance without Antibiotic Exposure
Source: Antimicrob Agents Chemother. 2017 Oct 24;61(11):e01495-17. doi: 10.1128/AAC.01495-17 (PMC5655081; doi:10.1128/AAC.01495-17)
Supplement: Supplemental material [file AAC.01495-17_zac011176671s1.pdf]

Supplementary information, Table S1, Materials and Methods

Evolution of antibiotic resistance without antibiotic exposure

Anna Knöppel[a*], Joakim Näsvall[a], and Dan I. Andersson[a#]

[a]Department of Medical Biochemistry and Microbiology, Uppsala University, Uppsala, 751 23, Sweden

[#]Address correspondence to Dan I. Andersson, Dan.Andersson@imbim.uu.se

[*]Present address; Department of Cell and Molecular biology, Uppsala University, Uppsala, Sweden.

Table S1. Mutations found through whole genome sequencing in the evolved *E. coli* and *S. enterica.*

Spec. media	Evol. pop	MIC[a]	Mutations[b]
Eco ancest[c]	DA5438		F^- λ^- *ilvG*$^-$ *rfb-50 rph-1*
Sal ancest[d]	DA6192		*treB*$^-$(K181fs; ΔA541)[d] *malP*(Ile458fs; Δ37 bp.) *malQ*(Leu96Arg) *malT*(-46+A)
Eco LB	DA31962		*gltP*(A-115T)
Eco LB	DA31963		*gltP*(A-115T) *arcA*(Phe79Leu) *arcA*(His40Pro) *trkH*(Pro94Thr)
Eco LB	DA31964		*gltP*(C-118ΔC) *rpoS*(Ile128Asn) *ompF*(C-128A [promoter region])
Eco LB	DA31965		*gltP*(A-115T) *rpoA*(Asn294Lys)
Eco LB	DA31966		*gltP*(A-115T) *rpoS*(Ile95Phe)
Eco LB	DA31967		*gltP*(A-115T) *ssuD*(syn P333 CCG --> CCA)
Eco LB	DA31968		*gltP*(A-115T)
Eco LB	DA31969		*gltP*(A-115T) *yrhA*(Asp128Ala) *sapF*(Ser184Leu)
Eco LB	DA31970		*gltP*(A-115T) *trkA*(Gln144His)
Eco LB	DA31971	3.20 (MEC)	*sapD*(Gly235Ser) *sapF*(Arg158Ser)
Sal LB	DA18273[e]		*treB*$^+$ (541insA)[f] *barA*(Leu273Gln) *fliH*(Thr11Pro) *nmpC/ompD*(del4aa)
Sal LB	DA18274[e]		*treB*$^+$ (541insT)[f] *barA*(Gln470*) *flhD*(Leu39Pro)
Sal LB	DA18275[e]		*treB*$^+$ (547insG)[f] *barA*(Gly455Cys) Δ*fliF*(*fliF* nt. 756 - *fliG* nt 25)
Sal LB	DA18276[e]		*treB*$^+$ (543insA)[e]f*barA*(ins ATT codon 402-403) *fliH*(Thr11Pro) *yjeP*(Ile893Phe) *sifB*(C-80A [promoter region])
Eco MH	DA29564[e]	0.40 (EM)	Δ(*rph*[620]-*pyrE*[-80]; 82bp deletion) *ftsQ*(ΔQ113) *ugpB*(Glu239Asp) *fimA*(promoter ON)[g] *fimA*(promoter::IS*5*) *fimH*(Gly87Val) *aceB-metC*(intergenic G-->C 59 nt after stop in *metC*)
Eco MH	DA29565[e]		*pyrE*(G-42C terminator) *ftsX*(ΔAla226) *gltP*(A-115T) *fimA*(promoter inversion)[g] *fimE*::IS*5* *fimD*(C-10A RBS) prophage *cps*-53(cut out, re-stores *attB*) *rrlC*(C2681A)
Eco MH	DA29566[e]	0.46 (EM)	*pyrE*(G-41ΔG terminator) *ftsI*(Thr424Ala) *fimA*(promoter inversion)[g] *fimE*::IS*5* *yobD*(R129Syn; CGT-->CGC) *flu*(Thr562Pro) *flu*(G-7T -10 box promoter)
Eco MH	DA29567[e]	0.44 (EM)	*pyrE*(G-41ΔG terminator) *ftsX*(fs; Δ10 bp) *entB*(fs; Δ11 bp) *fimE*::IS*1*
Eco MH	DA29568[e]		*pyrE*(G-41ΔG terminator) *fimA*(promoter inversion)[g] *flu*(Asp551Ala) *flu*(G-7T -10 box promoter)
Eco MH	DA29569[e]		*pyrE*(A-33ΔA del in polyU in terminator) *relA*(Ile295Lys) *lsrD*(Val160Gly) *fimA*(promoter inversion)[g] *fimE*(Ala123Val) *flu*(Leu500Pro)
Sal MH	DA18269		*malT*(Trp52Cys) *malE*(Trp184*) *rfbV*(Trp180Gly) *yeaG*(nt1592delG) *flgJ*(Gln102*) *stm2293*(Ser174fs) *rfaK*(Lys53fs) *glk*(Asn89fs) *fliH*(Thr11Pro)
Sal MH	DA18270		*malT*(Gln855*) *malQ*(Arg96Cys) *clpP*(C-211A) *fliP*(Gly186Asp) *fimZ*(T-518A) *stm0720*(Arg261His)
Sal MH	DA18271[e]		*malT*(Gln470*) Δ(*tnpA*[273]-*fliBCDST-amyA-yedDEF-fliEFGHIJKLMNOPQR-rscA-yodD-dsrA-yedP-stm1987-stm1988.S*[58]) 25810 nt
Sal MH	DA18272[e]	2.00 (FO)	*malT*(Δ27bp) Δ(*fliG*[633]-*fliHIJ-fliK*[159]; 3054bp) Δ(*fliM*[959]-*fliNOPQR-rscA-yodD-dsrA-yedP*[706]; 5162bp) *envC*(Δ nt. 1136-1153)
Eco M9 glyc	DA30755	>4 (EM)	*glpK*(Ala66Val) *rne*(Val213Leu) *kgtP*(Gly196fs)
Eco M9 glyc	DA30756	>4 (EM)	*glpK*(Ser58Arg) *glpK*(Asn325His) *rne*(Leu426Arg) *nusA*(Arg258Cys) dup(*rhsB-rhsA*; 145 kb)
Eco M9 glyc	DA30757	>4 (EM)	*glpK*(Asn325His) *glpK*(Arg34Ser) *relA*(Ser258Cys) *nusA*(Arg258Cys) *ppiB*(Asp149Asn) *ybfQ*(del10bp) *clpA*(Gln89Arg) *mdoH*(Val180fs) Δ(prophage e14 15204bp, precise excision, restores *icd*/*icdC*) *alsC*(Ile34Val)
Eco M9 glyc	DA30758		*glpK*(Ala19Ser) *cyaA*(Gln109Lys) *rhsB-rhsA*(dupl) *rrnC-rrnA*(dupl) *rpsJ*(Asp14Ala)
Eco M9 glyc	DA30759	>32 (RI); 2.67 (SM)	*glpK*(Ala354Val) *rpoC*(Arg1075Leu)
Eco M9 glyc	DA30760	2.17 (RI); 2.33 (SM)	*glpK*(Val8Phe) *rne*(Gly172Ser) *rpoB*(Arg12Cys)
Eco M9 glyc	DA30761		*glpK*(Ile238Thr) *rph*(G-41ΔG terminator) *rne*(Asp415Asn) *ybgP*(Lys64Gln)
Eco M9 glyc	DA30762	>32 (RI)	*glpK*(Arg189Ser) *glpK*(Arg157Gly) *glpK*(Ala19Ser) *rpoB*(His526Tyr) *rpoC*(Asn762His) dup(*rhsB-rhsA*; ~145kb)
Sal M9 glyc	DA20884		*glpK*(Gly232Asp) *nadR*(Gln134fs) *yehU*(Pro381Leu)
Sal M9 glyc	DA20885		*glpK*(Val8Ile) *nadR*(Leu251Phe)
Sal M9 glyc	DA20886		*glpK*(Gly231Asp) *nadR*(Gln134fs) *nadR*(Ala317Glu) *yecS*(G-20A)
Sal M9 glyc	DA20887		*glpK*(Arg34His) *nadR*(Trp157Arg)
Sal M9 glyc	DA20888		*glpK*(Val8Ile) *nadR*(Leu251Phe) *nadR*(Glu374*) *relA*(Arg280Cys)
Sal M9 glyc	DA20889		*glpK*(Arg34His) *nadR*(Trp157Arg)
Sal M9 glyc	DA20890		*nadR*(Gly290Asp) *relA*(Gly59fs) *yciM*(Tyr39Asp)
Sal M9 glyc	DA20891	3.17 (EM)	*glpK*(Gly234Asp) *relA*(Ile338Asn)
Eco M9 gluc	DA32272		*pyrE*(G-41ΔG terminator) *proQ*(Leu17Gln)
Eco M9 gluc	DA32273	2.07 (SM)	Δ(*rph*(620]-*pyrE*[-80]; 82bp deletion) *clpA*(Ser133Arg)
Eco M9 gluc	DA32274		*pyrE*(G-41ΔG terminator) *rpoB*(Leu671Met) *rpoS*(Ala130Glu)
Eco M9 gluc	DA32275		Δ(*rph*(620]-*pyrE*[-80]; 82bp deletion) *cysI*(Gly190Asp) *proQ*(Asp92fs)
Eco M9 gluc	DA32276	>32 (RI)	*pyrE*(G-42T terminator) *rpoB*(His526Tyr)
Eco M9 gluc	DA32277		Δ(*rph*(620]-*pyrE*[-80]; 82bp deletion) *proQ*(Asp92fs)
Eco M9 gluc	DA32278		*pyrE*(G-41ΔG terminator) *proQ*(Glu30*)
Eco M9 gluc	DA32279		*pyrE*(G-41ΔG terminator) *rpoB*(Val21Gly) *mreC*(Arg14Leu)
Sal M9 gluc	DA10876	5.80 (FO)	*iroN*(Ala162Thr) *pykF*(Arg73Leu) *rfbD*(Pro43fs) *emrR*(promoter) *rpoS*(Arg141Cys) dup(*rrnB-rrnE*)
Sal M9 gluc	DA10877	5.81 (FO)	*iroN*(Ser87Leu) *pykF*(Leu432Pro) *fes*(C-88T)/*fepA*(G-101A) *rfbC*(*184Ser) *clpA*(Thr354Met) *ytfG*(His154Pro) dup(*rrnB-rrnE*)
Sal M9 gluc	DA10878	5.11 (EM); 3.85 (FO)	*iroN*(Ser87Leu) Δ(*pykF*[1389]-*orf245*[27], 4819 nt) *fes*(T-82C)/*fepA*(A-107G) *rfbI*(G120syn; GGT-->GGG) *rfbC*(Arg60Ile) dup(*rrnB-rrnE*)
Sal M9 gluc	DA10879	4.44 (EM)	*iroN*(Ala162Val) *pykF*(Thr454fs) *fes*(G-87T)/*fepA*(C-102A) promoter region *rfbP*(Pro129fs) *ptsP*(Gln507*) dup(*rrnB-rrnE*)

[a] Average relative minimal inhibitory concentration (MIC) for populations that differed from wild-type (Fig. 2). EM = erythromycin, FO = fosfomycin, MEC = mecillinam, RI = rifampicin, and SM = streptomycin. For EM and RI, no inhibition zone was visible.

[b] Positions for mutations outside of coding sequences are given relative to the first nucleotide in the start codon of the downstream gene (negative numbers). For deletions, nucleotide positions are given for the beginning and end.

[c] In addition the mutations listed in the genotype of *E. coli* K12 strain MG1655 (U00096.3), our laboratory wild-type DA5438 contains the following mutations: *intF*(E203syn; GAG-->GAA) *gatC*$^+$(ΔC916-C917) *yohK*(L34S) *glpR*$^+$(+C151) *crl*$^+$(ΔIS*1*) *fadK*::IS*1* ΔIS*1*(*flhD-uspC* intergenic) ΔIS*5*(*ychE-oppA* intergenic) *rip321*(+CG; *gltP-yjcO* intergenic, 586 bp downstream of *gltP*, 55 bp downstream of *yjcO*).

[d] DA6192 additionally contains the following mutations compared to the published sequence of *S. enterica* serovar Typhimurium strain LT2 (AE_006468.2): Δ(*rhlB*[574]-*gppA*[391]; 1202 bp) *malP*(Ile458fs; Δ37 bp.) *malQ*(Leu96Arg) *malT*(-46+A) *rrsH*(C1529A) *brnQ*(Y227syn; TAC-->TAT) *fimH*(Gly61Ala) *dcuC*(Gly29Asp) *yea*G(Gly115fs; +G89) *rnb*(Trp234*) *stm1747*(Arg33Gln) *hnr*(V102Gly) *motA*(Pro271Leu) *mglA*(Val93Glu) *stm3633*(L188syn; TTA-->CTA).

[e] Sequenced isolated clone from the evolved population. All other whole genome sequencing was done on evolved populations.

[f] *S. enterica* ser. Typhimurium (our wild-type included) naturally carries a frameshift in *treB* (AAA AA[ΔA] ATG GGC; codons 180 - 183) and that is present in all sequenced *S. enterica* serovar Typhimurium. Our pseudo-revertants correct this frameshift to AAA AGA ATG GGC (DA18273), AAA ATA ATG GGC (DA18274), AAA AAA TGG GGC (DA18275), and AAA AAA ATG GGC (DA18276). The "real" wild-type sequence is AAA AAA ATG GGC.

[g] Promoter inversion. The *fimA* promoter is present on an invertible DNA element whose inversion is catalyzed by the recombinases FimE and FimB (1). The *fim* operon is only expressed when the *fimA* promoter is in the correct orientation).

Methods

Strains and media. *Escherichia coli* MG1655 (designated *E. coli* in the text) and *S. enterica* subsp. *enterica* serovar Typhimurium str. LT2 (*S. enterica*) were used as ancestors for the evolution experiments (Knöppel *et al.*, in preparation). The media used for serial passage were: LB – lysogeny broth (5 g yeast extract [Oxoid], 10 g Tryptone [Oxoid], 10 g NaCl L^{-1}), MH – Mueller Hinton (BD), and M9 minimal medium [5] supplemented with either 0.2% glucose, or 0.2% glycerol. All susceptibility testing was done using MH media (BD).

Serial passage procedure. Cultures of *E. coli* and *S. enterica* were started from single colonies in four different media (LB, MH, $M9^{gluc}$, and $M9^{glyc}$; Knöppel *et al.*, in preparation). In 24 h intervals, the independent lineages were serially passaged by 1,000-fold dilution in 1 or 1.5 ml batch cultures, using 10 ml tubes (Sarstedt) (Fig. 1). The bottlenecks under these conditions were approximately 5×10^6 bacteria per transfer for the complex media and 1×10^6 for the minimal media. The approximate numbers of generations for each of the 8 conditions are shown in Fig. 2.

Susceptibility tests. Susceptibility of the evolved populations to different antibiotics (ampicillin, chloramphenicol, ciprofloxacin, erythromycin, fosfomycin, mecillinam, nitrofurantoin, streptomycin, and tetracycline) was determined using Etests according to the manufacturer's instructions (Liofilchem s.r. l., Italy [mecillinam and rifampicin] bioMérieux, Marcy, l'Êtoile, France [all other]).

Prior to the tests, an aliquot of the frozen population was resurrected and grown in MH overnight. The cultures were diluted 20-fold in PBS and used for the tests, and the test results were read after approximately 18 h. The fold change was calculated by dividing the test results for the evolved populations by the results for the un-evolved ancestors performed at the same day. Tests that showed at least a two-fold difference compared to the ancestor were repeated at least three times. Populations that consistently showed a higher MIC than the ancestor, and with average increases that were two-fold or higher were considered less susceptible than the ancestor. Similarly, to be considered more susceptible than the ancestor, the population had to show a lower MIC in all repeats, and have an average relative MIC of at most 0.5.

Whole genome sequencing and sequence analyses. The sequencing was performed on the evolved populations (and in some cases on isolated clones). Briefly, genomic DNA was prepared using Genomic Tip 100/G (Qiagen) or MasterPure™ (Epicentre), and sequencing was done using Illumina HiSeq (at BGI, Beijing, China) or Illumina MiSeq. All bioinformatics analyses used CLC Genomics Workbench (CLC bio, Aarhus, Denmark). SNPs and indels were called using the low frequency variant detection tool, and larger duplications or deletions were searched for both by visual scanning of the assembled read depth and by the structural variants detection tool. Mutations with frequencies higher than 10% were considered real if they were present in high quality reads. Mutations identified are listed in Supplementary Table S1.

References

1. Holden N, Blomfield IC, Uhlin BE, Totsika M, Kulasekara DH, Gally DL. 2007. Comparative analysis of FimB and FimE recombinase activity. Microbiology 153:4138–4149.
